# Evaluating the Combined Anticancer Response of Checkpoint Inhibitor Immunotherapy and FAP-Targeted Molecular Radiotherapy in Murine Models of Melanoma and Lung Cancer

**DOI:** 10.3390/cancers14194575

**Published:** 2022-09-21

**Authors:** Kathleen M. Capaccione, Mikhail Doubrovin, Brian Braumuller, Dev Leibowitz, Nikunj Bhatt, Fatemeh Momen-Heravi, Andrei Molotkov, Michael Kissner, Kimberly Goldner, Mark Soffing, Alessandra Ali, Akiva Mintz

**Affiliations:** 1 Division of Nuclear Medicine and Molecular Imaging, Department of Radiology, Columbia University Irving Medical Center, New York, NY 10032, USA; 2 College of Dental Medicine, Columbia University Irving Medical Center, New York, NY 10032, USA; 3 Flow Cytometry Core Facility, Columbia Stem Cell Initiative, Columbia University Irving Medical Center, New York, NY 10032, USA

**Keywords:** molecular targeted radiotherapy, immunotherapy, melanoma, lung cancer

## Abstract

**Simple Summary:**

Although newer cancer medicines that help the immune system recognize and attack cancer cells have improved responses to therapy, most patients ultimately have cancer recurrence. Additional therapies and therapy combinations are needed so that responses can last longer or indefinitely. Molecular targeted radiotherapy is another kind of therapy that targets radioactive particles directly to cancer in the hopes of killing cancer cells to stop tumor growth with limited side effects. Prior studies have shown that targeted radiotherapies activate the immune system and can work together with immunotherapy to improve response. Here, we tested a promising new therapy targeting fibroblast activation protein (FAP) with a therapeutic radionuclide ^177^Lu alone and with immunotherapy in mouse models of melanoma and lung cancer. The FAP-targeted radiotherapy reduced tumor growth in both models and melanoma, resulting in tumor regression. We saw increased tumor cell death in dual-treated tumors. We also found that myeloid cells were affected by the combined therapy to a greater degree than the additive effect of either therapy. These results demonstrate that this is a promising new therapy regimen and requires further preclinical and clinical study to better understand the molecular mechanisms underpinning response.

**Abstract:**

Immunotherapy has dramatically improved outcomes for some cancer patients; however, novel treatments are needed for more patients to achieve a long-lasting response. FAP-targeted molecular radiotherapy has shown efficacy in both preclinical and clinical models and has immunomodulatory effects. Here, we studied if combined immunotherapy and radiotherapy could increase antitumor efficacy in murine models of lung cancer and melanoma and interrogated the mechanisms by which these treatments attenuate tumor growth. Using LLC1 and B16F10 murine models of lung cancer and melanoma, respectively, we tested the efficacy of ^177^Lu-FAPI-04 alone and in combination with immunotherapy. Alone, ^177^Lu-FAPI-04 significantly reduced tumor growth in both models. In animals with melanoma, combined therapy resulted in tumor regression while lung tumor growth was attenuated, but tumors did not regress. Combined therapy significantly increased caspase-3 and decreased Ki67 compared with immunotherapy alone. Flow cytometry demonstrated that tumor-associated macrophages responded in a tumor-dependent manner which was distinct in animals treated with both therapies compared with either therapy alone. These data demonstrate that ^177^Lu-FAPI-04 is an effective anticancer therapy for melanoma and lung cancer which mediates effects at least partially through induction of apoptosis and modulation of the immune response. Translational studies with immunotherapy and ^177^Lu-FAPI-04 are needed to demonstrate the clinical efficacy of this combined regimen.

## 1. Introduction

Extensive progress in the fight against cancer has been made in the past several decades, in some cases transforming it from a deadly disease to a controlled illness requiring ongoing treatment. The advent of immunotherapy represented one of the greatest advances in anticancer therapy. In 2010, Hodi et al. published their Phase III trial of the anti-CTLA-4 immunotherapy ipilimumab, demonstrating that approximately 1 in 5 patients achieved a lasting response [1]. The Checkmate 067 trial studied the effects of combined ipilimumab and nivolumab and found the overall response rate was 58% in the combination group versus 19% in the ipilimumab alone group [2]**,** resulting in FDA approval of the combined regimen as first-line therapy for melanoma [3]. Checkpoint inhibitors were subsequently studied for the treatment of solid tumors, including non-small cell lung cancer (NSCLC) [4,5,6]. The PD-1 inhibitor nivolumab was initially approved in 2015, and a subsequent study evaluating nivolumab and ipilimumab demonstrated a 23.3-month response compared to a 6.2-month response with chemotherapy [6]. Given these initial successes and subsequent studies confirming the survival benefit of immunotherapy, they are now first-line therapy for NSCLC, except in specific cases of patients with specific targetable driver mutations [7].

Despite these advances, many patients with cancer do not achieve a lasting response. External beam radiotherapy has long been a pillar of NSCLC treatment. Recently, molecular targeted radiotherapies have been developed against tumor-restricted markers in other cancers and have demonstrated efficacy in preclinical models and clinically [8,9,10]. Several molecular targeted radiotherapies have been studied in preclinical models of melanoma, while, to our knowledge, no studies have demonstrated the efficacy of molecular targeted radiotherapy for the treatment of NSCLC [8,11].

Fibroblast activation protein (FAP) is a serine protease expressed in cancer-associated fibroblasts (CAF) cells that ubiquitously infiltrate tumors and form part of the tumor microenvironment. FAP is expressed in a large number of cancers, and research in melanoma has shown that FAP is expressed in cancer-associated fibroblasts yet is not expressed in cancer cells themselves [12]. Targeting CAFs is a rational anticancer strategy given that CAFs are ubiquitous within tumors to provide support for cancer cells [13]. FAP is an attractive target for molecular radiotherapy, given its low-level expression in normal tissue [14]. Lindner et al. developed a series of quinolone-based theranostic ligands targeting FAP and characterized their binding and pharmacokinetics [15]. In this study, the group tested the quinolone-based compounds in FAP-expressing HT-1080 cells. Compounds were tested for internalization, and those with internalization values greater than 90% were advanced to the next phase of testing. Subsequent studies evaluated accumulation at different time points, and compounds not demonstrating good accumulation at 1, 4, and 24 h were not advanced to the next stage of testing. The remaining compounds were tested for target specificity using human embryonic kidney cells, and ultimately FAPI-02 and FAPI-04 demonstrated the best overall binding characteristics and were therefore chosen for use in future studies [15]. Many subsequent preclinical [16,17] and clinical [18,19,20] studies have further evaluated the efficacy of these molecules for imaging and therapy applications. Continued development of FAPI agents led to the production of FAPI-46 which authors demonstrated to have increased tumor retention time compared with FAPI-04 [21] as well as FAPI-74, which has a NOTA moiety, allowing it to be labeled with either ^68^Ga or ^18^F via complexing with aluminum, a key advantage for increasing production scale [22,23].

Recently, the combination of ^64^Cu-FAPI-04 and ^225^Ac FAPI-04 in a mouse model of pancreatic cancer showed uptake within tumors and significant tumor suppression [17]. Several studies have evaluated the combined effects of molecular targeted radiotherapy and immunotherapy because of the potential for enhanced efficacy mediated by the abscopal effect [8,10]. Studies evaluating the combined effects of molecular targeted radiotherapy and immunotherapy in other cancers have demonstrated excellent potential for increased response to therapy and potential lasting response; however, data on lung cancer are lacking [9,24,25,26]. ^177^Lu is a therapeutic radionuclide that decays principally through β minus decay, which causes double-strand DNA breaks leading to irreparable cell damage and, ultimately, cell death [27]. Here, we evaluate the effects of checkpoint inhibitor immunotherapy and ^177^Lu-FAPI-04 individually and in combination in melanoma and lung cancer models to provide a comparative analysis of tumor types and the first data on lung cancer and the molecular effects of combined radiotherapy and immunotherapy.

Prior studies have demonstrated that tumor-infiltrating lymphocyte [28] and myeloid lineage tumor-infiltrating cell [29] populations mediate immunotherapy response and have prognostic value. Dendritic cells serve as antigen-presenting cells and mediate the antitumor T-lymphocyte immune response, fundamentally bridging the gap between innate and adaptive immunity. They also contribute to the cytotoxic immune response via natural killer (NK) cell activation [30]. Myeloid cells within the tumor microenvironment produce inflammatory mediators such as IL-6 and TNF-a, promoting tumor growth, inflammation, and angiogenesis [31]. The effect of radiotherapy on tumor-associated macrophages (TAMs) is complex. Klug et al. demonstrated that low-dose irradiation results in the induction of TAMs necessary for immune-mediated tumor rejection. They demonstrated that CD8+ T-cells could establish a protumor environment through TAMs, which can be abrogated via low-dose irradiation [32]. Ratios of M1 versus M2 macrophages have been associated with outcomes, and M2 macrophages are principal resident cells within the tumor microenvironment, regulating the balance between immunosuppression and immune activation, ultimately facilitating response to immunotherapy. We investigated the effects of FAP-targeted radiotherapy alone and in combination with immunotherapy on TAM populations to better understand how combined therapy may modulate response to therapy. 

## 2. Materials and Methods

### 2.1. Animal Model

C57BL/6 male mice were obtained from Taconic Biosciences and maintained by the Columbia Institute for Comparative Medicine under IACUC protocol AC-AAAT9470 (approved 9/12/17). We established models of melanoma and lung cancer using B16F10 and LLC1 cells, respectively, with subcutaneous tumors implanted in the region overlying the right shoulder. Cell lines were obtained from ATCC (Manasses, VA, USA). Animals were assessed, the tumors’ greatest dimension (in mm) was measured using calipers, and weight was recorded every 2–3 days. Animals were euthanized when tumors reached 20 mm or greater or if they ulcerated through the skin. The experiment was prespecified to end on day 21, and all remaining animals were euthanized at that time. 

B16F10 murine melanoma cells were cultured and expanded in DMEM (Gibco, Waltham, MA, USA), and LLC1 murine lung cancer cells were cultured and expanded in RPMI (Gibco, Waltham, MA, USA), as previously described [33]. Cell lines were visually assessed for mycoplasma and growth rates compared to those in the literature; if abnormal, cells were tested for mycoplasma. Media contained 10% FBS, 1% Antibiotic-Antimycotic (ThermoFisher Scientific, Waltham, MA, USA), and 1% L-glutamine (ThermoFisher Scientific, Waltham, MA, USA). Cells for injection were washed and then harvested using Trypsin EDTA (Gibco, Waltham, MA, USA) and counted manually using a hemocytometer. B16F10 or LLC1 cells were injected subcutaneously into the posterior tissues overlying the right shoulder 1:1 with growth factor reduced Matrigel (Corning, Corning, NY, USA). 

Four groups of animals were used for each model in this study: untreated control, immunotherapy treated, radiotherapy treated, and combined immunotherapy and radiotherapy treated. Mice were approximately 4–6 weeks old, with an average weight of 20 g. Animals were randomized to ensure a similar distribution of tumor sizes among groups prior to treatment. The study was not blinded, given that animals underwent different treatments administered by investigators. Tumor size in millimeter (mm) was the primary outcome measure. Animals were housed in a vivarium for the duration of the study and were given access to unlimited regular food and water.

Mice were ear-tagged, and tumor size and animal weight were measured every 1–2 days. Animals were euthanized at 21 days, when tumors were greater than 2 cm in any dimension or if tumors had ulcerated or were about to ulcerate through the skin. At this time, tumors were collected to process for downstream applications. In animals that had a partial or complete response, the tissues in the region of tumor injection were extracted for analysis of residual cells.

### 2.2. Immunotherapy Treatment 

Animals in the immunotherapy (IT) treatment arms were treated with a combination of 200 ug per animal anti-murine CTLA-4 and 200 ug anti-murine PD-1 on days 12, 15, and 18, as per prior studies [34].

### 2.3. ^177^ Lu-FAPI-04 Molecular Targeted Radiotherapy

Prior to the administration of ^177^Lu-FAPI-04 molecular targeted radiotherapy, ^68^Ga-FAPI-04 PET scans were performed in both tumor types to demonstrate adequate ^68^Ga-FAPI-04 uptake within tumors. ^177^LuCl3 was received (Eckert & Ziegler Nuclitec GmbH) and used as such. ^177^Lu 10.3 mCi was equilibrated to pH 4.5 with 0.1 M sodium acetate buffer, followed by the addition of 25 µL of sodium ascorbate (pH 4.5) and 10 μg FAPI-04. The reaction was incubated at 90°C for 25 min in a thermomixer (600 rpm). The formation of ^177^Lu-FAPI-04 was monitored by radio-TLC using Varian ITLC-SG strips developed with 50 mM EDTA (pH 5.0) as the mobile phase. In this system, free ^177^Lu moved near to solvent front (Rf = 0.9 to 1.0) whereas ^177^Lu-FAPI-04 remained near the origin (Rf = 0.0 to 0.1). The ^177^Lu-FAPI-04 was further confirmed by reverse-phase HPLC (RP-HPLC) using a Phenomenex Luna C18(2) column (5 μm, 250 × 4.6 mm) eluted with a mobile phase consisting of 0.1% TFA/H2O (solvent A) and 0.1% TFA/acetonitrile (solvent B), and a gradient consisting of 1% B at 0 min to 25% B in 15 min at a flow rate of 1 mL/min. Formed ^177^Lu-FAPI-04 (~97% pure) was used without further purification. The specific activity of formed ^177^Lu-FAPI-04 was calculated to be 1.0–1.1 mCi/ μg (870–950 mCi/ μmol). Animals were treated with 1.5 mCi ^177^Lu-FAPI-04 radiotherapy (RT) on day 13 of the experiment based on prior studies demonstrating efficacy at this dose against an animal model of sarcoma [35].

All experimental procedures involving radioactive material were performed in accordance with guidelines of the Nuclear Regulatory Committee, the Columbia University Radiation Safety Committee, and the approved IACUC protocol governing the study with a specific radiation safety appendix approved by the Columbia University Radiation Safety Officer. In short, all personnel underwent extensive radiation safety training prior to inclusion in the protocol. All animals in this study were housed in a dedicated satellite vivarium once activity had been administered. Surfaces such as lab bench tops and door handles were coated with disposable protective material for any time that animals were outside the satellite vivarium to prevent contamination of the laboratory. Personal protective equipment was used during all radioactive handling, including double gloves, gowns, and foot coverings. All used disposable materials such as animal bedding and gloves were collected in decay-in storage barrels and allowed to decay for 10 half-lives before disposal in standard waste streams. Radioactive animal carcasses and byproduct materials were treated similarly. All nondisposable equipment, such as animal cages and dissection instruments, were decayed in storage for 10 half-lives, after which they were cleaned and put back into use. A radioactive survey was performed daily, both by investigators and the laboratory manager, to ensure that no contamination had taken place.

### 2.4. Immunofluorescence

Tumors were extracted after mice euthanasia and preserved in 4% paraformaldehyde (PFA) overnight, then transferred to 30% sucrose for a minimum of 24 h. Subsequently, they were embedded in OCT medium (Tissue-Tek^®^, Torrance, CA, USA) and sectioned on a cryostat in 10 um sections on poly-L-lysine coated slides.

Immunofluorescence (IF) was performed according to the following protocol: frozen sections were washed twice with PBS to rehydrate. Subsequently, they were blocked with 5% BSA for 1 h prior to the primary antibody. Slides were washed 5 times with PBS+0.5% BSA (PBB), then primary antibody was added at the appropriate concentration in PBB. Twelve hours later, slides were again washed 5 times with PBB, then goat anti-rabbit Alexa488 was added for 1 h and incubated at room temperature. Slides were then washed 5 times with PBB followed by 5 times with PBS before adding DAPI at 1:10,000 concentration. Slides were washed 3 times with PBS, then VectaShield and a coverslip were added prior to imaging. The following antibody concentrations were used for staining: anti-Casp-3 (ab2303) 1:200; anti-Ki67 (ab15580) 1:100. 

### 2.5. Flow Cytometry

The gating strategy for our flow cytometry experiments is shown in Table 1. Tumors were manually dissociated into a single cell suspension, RBC lysis was performed, and preserved in 2% PFA for subsequent staining. For staining, cells in suspension were washed and then blocked in 2% fetal bovine serum (FBS) for 1 h prior to staining to prevent nonspecific binding. Subsequently, cells were washed and resuspended in 100 uL flow cytometry staining buffer. Antibodies were added at a concentration of 1:100 to the cell suspension and incubated for 20 min before washing. Cells were then resuspended in 300 uL flow cytometry staining buffer for analysis. The gating strategy is presented in Table 1. Stained cells were analyzed using a BD FACSCelesta™ Cell Analyzer (BD Biosciences, Franklin Lakes, NJ, USA). 

### 2.6. Statistical Analysis

Statistical analysis was performed using Microsoft Excel v.16.48 and FACsDiva software. ANOVA was used to evaluate if significant differences existed among the groups for IF and flow cytometry. In cases where there were significant differences, groups were further analyzed by Student’s t-test between each group to identify pairs with significant differences. A *p* < 0.05. was considered statistically significant.

## 3. Results

### 3.1. Molecular Targeted ^177^Lu-FAPI-04 Radiotherapy and Immunotherapy Attenuate Tumor Growth in a Tumor-Dependent Manner

**Figure 1:**
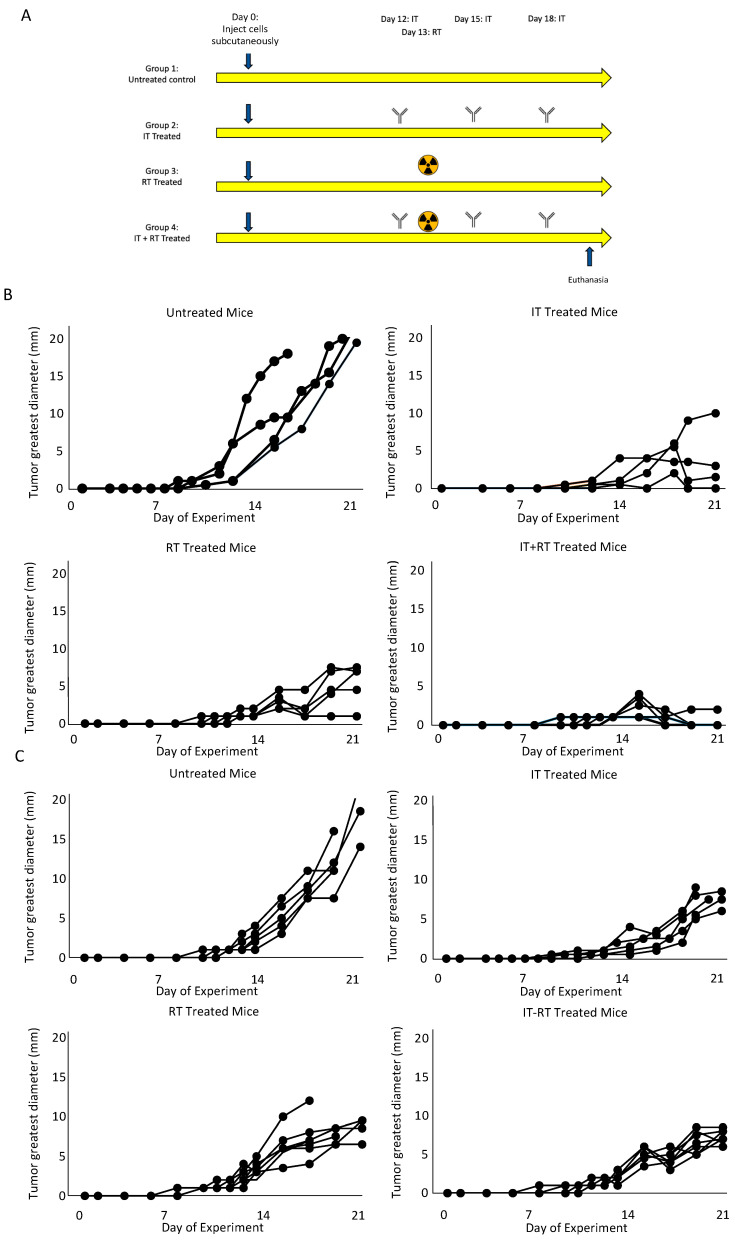
Experimental plan and tumor growth curves. (**A**) demonstrates the treatment regimen used in this study. Tumor growth curves for melanoma (**B**) and lung cancer (**C**) for each condition demonstrated responsiveness to both immunotherapy and radiotherapy.

Figure 1A demonstrates the study design. We followed tumor growth over time to assess if ^177^Lu-FAPI-04 radiotherapy attenuated tumor growth and found that it significantly decreased tumor growth in both mouse models of melanoma (Figure 1B) and lung cancer (Figure 1C). Supplemental Figure S1A,B shows consolidated growth curves for each of the treatment groups in melanoma and lung cancer. The effect of immunotherapy combined with radiotherapy was different in the different tumor types; in melanoma immunotherapy resulted in partial regression in the majority of tumors; however, in lung cancer, it attenuated tumor growth although tumors did not show regression. In melanoma, there was a statistically significant reduction in tumor growth compared to control with immunotherapy (*p* = 0.00004), radiotherapy (*p* = 0.00002), and the combined regimen (*p* = 0.00002). The combined regimen resulted in significantly decreased growth compared with radiotherapy alone (*p* = 0.005) and trended toward, but did not achieve, significance compared with immunotherapy alone (*p* = 0.09). In lung cancer, there was a statistically significant reduction in tumor growth compared with control with immunotherapy (*p* = 0.0008), radiotherapy (*p* = 0.0005), and the combined regimen (*p* = 0.00001). The combined regimen resulted in significantly decreased growth compared with radiotherapy alone (*p* = 0.05) but was not significantly different from immunotherapy alone (*p* = 0.88).

### 3.2. Molecular Targeted ^177^Lu-FAPI-04 Radiotherapy Results in Increased Apoptotic Cell Death and Attenuated Cell Cycling

**Figure 2:**
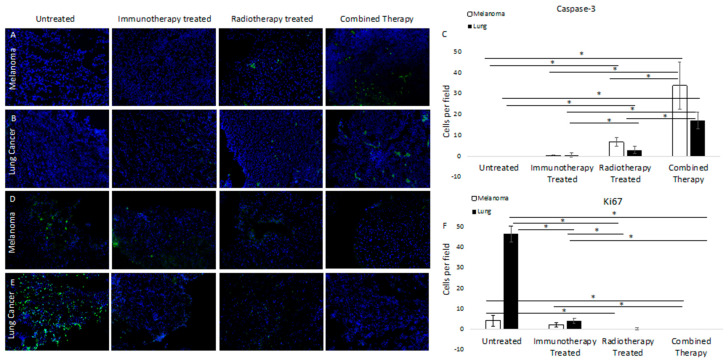
Immunofluorescence staining of untreated and treated tumors for caspase-3 in melanoma (**A**) and lung cancer (**B**) demonstrated increased staining in samples treated with either immunotherapy or FAP-targeted radiotherapy, with markedly increased caspase-3 activity in tumors treated with both. (**C**) Quantifies positive cells per field and demonstrates significantly increased caspase-3 staining for the combined therapy group compared to untreated, immunotherapy, or radiotherapy treated samples (*p* = 0.001, 0.003, and 0.001 for melanoma and *p* = 0.0002, 0.0007, and 0.002 for lung cancer, respectively). Conversely, staining for Ki67 in melanoma (**D**) and lung cancer (**E**) demonstrated significant differences in staining of untreated samples, with decreased staining in samples treated with either therapy and essentially no Ki67 staining in tumors treated with dual therapy. (**F**) demonstrates quantification of positive cells per field and significantly decreased staining for the combined therapy group compared with untreated and immunotherapy-treated samples (*p* = 0.02, 0.003, respectively). For lung cancer, there were significant differences in Ki67 staining in the combined therapy group compared with untreated and immunotherapy-treated samples (*p* = 0.0000007 and 0.0005, respectively). Significant differences are marked with a line with an asterisk.

We assessed apoptotic cell death by anti-caspase-3 (casp-3) immunofluorescence. Results for murine models of melanoma and lung cancer were concordant, demonstrating no staining in untreated tumor samples, minimal caspase-3 staining in immunotherapy-treated tumors, and mild caspase-3 staining in ^177^Lu-FAPI-04 radiotherapy-treated tumors. In both cases, there was a significantly higher caspase-3 signal in the combined therapy groups than in either alone (Figure 2A,B, quantified in C). Quantification of positive cells per field demonstrated significantly increased caspase-3 staining for the combined therapy group compared with untreated, immunotherapy, or radiotherapy-treated samples (*p* = 0.001, 0.003, and 0.001 for melanoma and *p* = 0.0002, 0.0007, and 0.002 for lung cancer, respectively). 

To assess cell cycling, we performed anti-Ki67 immunofluorescence. Results for murine models of melanoma and lung cancer were substantially concordant and demonstrated a greater degree of staining in untreated samples than in treatment groups. For both models, there was mild Ki67 staining in the immunotherapy-treated groups and minimal in the ^177^Lu-FAPI-04 radiotherapy group. There was no Ki67 staining in the combined therapy samples from either group (Figure 2D,E; quantified in F). Quantification of positive cells per field demonstrated significantly decreased staining for the combined therapy group compared with untreated and immunotherapy-treated samples in melanoma (*p* = 0.02, 0.003, respectively). In lung cancer, there were significant differences in Ki67 staining in the combined therapy group compared with untreated and immunotherapy-treated samples (*p* = 0.0000007 and 0.0005, respectively).

### 3.3. ^177^ Lu-FAPI-04 Radiotherapy and Immunotherapy Induce Changes in Conventional Dendritic Cell lineage I and II Populations in Melanoma

**Figure 3:**
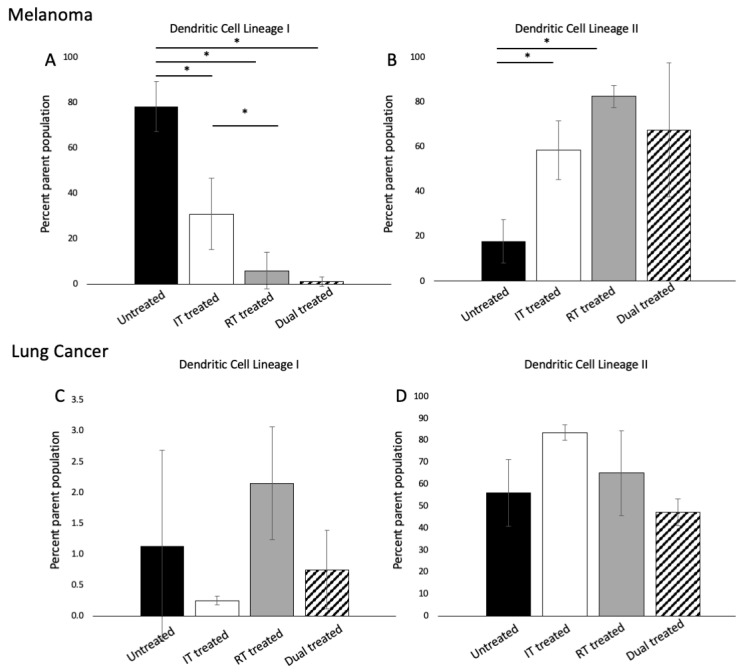
Flow cytometric analysis of conventional dendritic cell lineage I and II cells in melanoma (**A**,**B**) and lung cancer (**C**,**D**) demonstrated that response to therapy was tumor-dependent; in melanoma, there were significant differences among both populations based on treatment group whereas these differences were not observed in lung cancer (*n* = 3 per group). Significant differences are marked with a line with an asterisk. (Dendritic cells: CD3^–^/CD19^–^/CD11c^+^/MHCII^+^; conventional DC lineage I: CD3^–^/CD19^–^/CD11c^+^/MHCII^+^/XCR1^+^/CD11b^–/low^, conventional DC lineage II: CD3^–^/CD19^–^/CD11c^+^/MHCII^+^/XCR1^–^/CD11b^+^).

We sought to identify changes in the immune cell populations which might portend lasting responses to therapy. In melanoma and lung cancer, there was no change in the overall dendritic cell populations based on treatment group (*p* > 0.05); however, in melanoma, there were statistically significant differences among conventional dendritic cell lineage I (*p* = 0.00006) and II (*p* = *0*.02) populations by ANOVA (Figure 3A,B). In lung cancer, differences among groups were not significant (Figure 3C,D). Supplemental Figure S1 presents an example of a flow gating strategy for dendritic cell populations.

### 3.4. ^177^ Lu-FAPI-04 Radiotherapy and Immunotherapy Modulate Myeloid Lineage Populations in a Tumor-Dependent Manner

**Figure 4:**
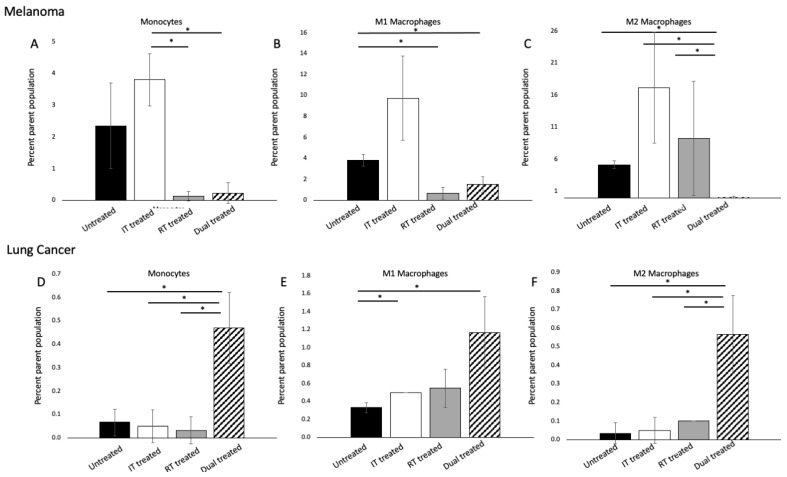
Flow cytometric analysis of myeloid lineage cells. In melanoma, (**A**) significant differences were seen among monocyte populations. In (**B**), M1 macrophages were significantly reduced in dual-treated samples compared with untreated tumors, and in (**C**), the M2 population was abrogated with combined therapy. In lung cancer, combined therapy led to significant increases in both monocytes, M1, and M2 macrophages compared with either treatment alone (**D**–**F**) (*n* = 3 per group). Significant differences are marked with a line with an asterisk. (M1 macrophages: CD80^+^/CD11b^+^; CD11B^+^/CD206^+^). Supplemental Figure S2 presents an example flow gating strategy for myeloid cell populations.

We sought to identify changes in tumor-associated macrophage populations resulting from ^177^Lu-FAPI-04 radiotherapy with and without immunotherapy compared to untreated tumors. In our murine model of melanoma, there were significant differences in the monocyte populations among treatment groups (*p* = 0.037) (Figure 4A). Evaluating M1 macrophages, we found that both radiotherapy and combined immunotherapy and radiotherapy treatment significantly reduced this population compared with untreated tumors (*p* = 0.009 and *p* = 0.02, respectively) (Figure 4B). M2 macrophages exhibited a paradoxical response in dual-treated tumors, with modest increases in M2 macrophages resulting from each therapy alone but a near complete abrogation of M2 macrophages when combined (*p* = 0.0001) (Figure 4C).

There were profound differences in the myeloid cell response in lung cancer compared with melanoma. For monocytes, M1, and M2 macrophages, dual treatment with immunotherapy and FAP-targeted radiotherapy resulted in a statistically significant increase in cell populations compared with untreated samples or either therapy alone (monocytes: *p* = 0.01; M1 macrophages: *p* = 0.02; M2 macrophages: *p* = 0.01) (Figure 4D–F). Importantly, the increase in cell populations seen in dual-treated samples was significantly more than the additive effects of the other two therapies, implying that the therapies increased each other’s induction of myeloid lineage cells. Given the marked differences between melanoma and lung cancer, it is clear that these changes are highly tumor and context-dependent and warrant further investigation.

## 4. Discussion

Molecular targeted radiotherapy has shown great potential to provide a therapeutic option for patients who do not achieve a lasting response with standard care therapy [36,37]. Here, we demonstrate that ^177^Lu-FAPI-04 radiotherapy effectively arrests tumor growth in murine models of both melanoma and lung cancer. These findings concur with prior data demonstrating the efficacy of ^225^Ac-FAPI-04 in a preclinical model of pancreatic adenocarcinoma [17], yet provide new data showing, for the first time, that targeted radiotherapy is an effective anticancer strategy in lung cancer. Here, we additionally evaluated the effect of this treatment with and without standard immunotherapy. Our data suggest that response to combined therapy is highly tumor- and context-dependent but leads to significant modulation of TAM populations that could produce clinically relevant immunologic changes. The concordant results of Ki67 immunofluorescence suggest that increased apoptotic cell death is at least in part responsible for the reduction in tumor volume seen in dual-treated tumors in both melanoma and lung cancer models. 

Macrophages are the most abundant tumor-infiltrating cells, and the M1 and M2 macrophage populations in solid tumors play a key role in promoting pro versus antitumor microenvironmental conditions [38]. Macrophages exhibit a high degree of plasticity with interleukin(IL)-12 and IL-18 or activated Toll-like receptors (TLRs) and IL-4, IL-10, and IL-13, resulting in M1 versus M2 cell polarization, respectively [39]. M1 macrophages promote innate host defenses and tumor cell killing via the production of inflammatory cytokines and reactive oxygen species (ROS) [40]. M2 macrophages produce anti-inflammatory cytokines, which promote a microenvironment conducive to tumor growth [41]. When the M1/M2 balance favors the M2 phenotype, this cell population promotes tumorigenesis through multiple pathways, including tumor growth, tumor cell migration, and invasion, inactivation of T-cells, enhancing resistance to chemo- and radiotherapy, EMT and tissue remodeling, angiogenesis, and others [39]. Despite progress in understanding the role of macrophage populations in tumor regulation, much work is yet to be done to allow for the manipulation of these cell populations towards an antitumor microenvironment that would translate to clinical response.

Inefficient T-cell recruitment can be a major impediment to effective immune activation and antitumor activity in the setting of immunotherapy. Prior research has demonstrated that low-dose external beam gamma irradiation can prime the tumor microenvironment so as to tip the M1/M2 macrophage balance favorably toward effective T-cell recruitment [32]. Here, we provide evidence that molecular targeted radiotherapy can function similarly to alter the M1/M2 macrophage ratio to create a permissive environment for effective T-cell recruitment, enhancing the efficacy of immunotherapy. Classically, IL-4 and IL-13 promote the development of the M2 phenotype, which suppresses antitumor immunity via the production of cytokines, arginase, and TGF-β [42]. In our mouse model of melanoma, while treatment with either immunotherapy or FAP-targeted radiotherapy increased the M2 macrophage population, combined therapy resulted in a near complete obliteration of this population. Prior research investigating the effects of combing radiation adjunctive therapies suggests that these agents can modify the recruitment and phenotype of macrophages and alter tumor microenvironmental modifiers. A study in a rat model of colorectal cancer evaluated the effect of administering toll-like receptor 4 (TLR4) lipopolysaccharide (LPS) or toll-like receptor 5 (TLR5) agonist flagellin after radiation. They found that these agents drove the tumor microenvironment toward the pro-M2 microenvironment via an increase in Arg and CD163 [43]. Prior work has also demonstrated that radiation induces dynamic changes in both cell populations and cell cytokines [44,45]. In our study, it is likely that dynamic changes occurred in cell populations and cytokines throughout the course of the experiment, and our analysis represents a snapshot of these factors at the predetermined study endpoint. Future work will evaluate the dynamic changes by sampling tumors throughout the study duration, allowing for the establishment of the temporal effects of radiation on macrophage populations.

Paradoxically, M2 macrophages were increased in lung tumors treated with combined therapy. Sumitomo et al. investigated M2 macrophages in 160 patients with resected non-small-cell lung cancer and found that stromal density of M2 macrophages was associated with Ki67 proliferation index, invasion, lymph node metastasis and stage of disease, overall portending a poorer prognosis [46]. In contradistinction to melanoma, where the decrease in M2 macrophages was associated with tumor regression, in dual-treated lung tumors, there was an increased M2 macrophage population and tumor growth was reduced; however, tumor regression was not seen. 

Our results demonstrate that response to combined targeted radiotherapy and immunotherapy is tumor dependent. Tumor microenvironmental conditions have previously been described as “hot” versus “cold” to reflect whether there is an ambient proinflammatory microenvironment that is replete with T-cells available to combat tumor cells [47]. Previous studies evaluating the B16F10 cell line and parent B16 cell line have characterized it as having a cold tumor microenvironment; however, these studies have also characterized the cell line as minimally responsive to checkpoint inhibitor immunotherapy [48,49]. While some studies have demonstrated that B16F10 tumors are refractory to checkpoint inhibitor immunotherapy, our data, as well as that of others [50,51]**,** has demonstrated partial response with this therapy, calling into question whether the B16F10 microenvironment can truly be considered “cold” or if there are other factors at play that contribute to the response to therapy. Most likely, there is a complex interplay of pro and antitumor signals which respond dynamically to the surrounding environment and treatments. Less is known about the immune environment of the LLC1 cell line; however, insights can be derived from a study by Pullamsetti et al. studying pulmonary hypertension arising in the setting of lung cancer. Using the LLC1 murine model, as well as two other murine models of lung cancer in comparison to an immunodeficient model, they found that immune cells were responsible for enhanced migration, evasion of apoptosis, and upregulation of vascular cells which led to pulmonary hypertension. These data strongly imply that immune cells play an important role in the LLC1 tumor microenvironment and are responsible, at least in part, for the differential changes in the different therapy groups observed in this study [52].

Similar to macrophages, dendritic cells play an important role in antigen presentation and initiation of the immune response, bridging the gap between innate and adaptive immunity. Conventional dendritic type I cells are traditionally recognized as responsible for anticancer immunity [53]; however, studies have shown that in certain tumor types and with certain therapies, dendritic type II cells can also play an important role [54]. Using a B16F10 murine model of melanoma, Binneswies et al. studied myeloid cells in tumor-draining lymph nodes and found that type II dendritic cells present tumor-derived antigens to conventional CD4+ T-cells and their abundance correlated with conventional CD4+ T-cells and patient survival. Our data demonstrated that radiotherapy significantly increased the type II dendritic cell population; however, this increase was attenuated when combined with immunotherapy. This suggests a complex interplay of immune activation, which likely depends not only on tumor type but also on the timing of therapies. Here, radiotherapy was given one day after the first immunotherapy dose, similar to prior studies [8,34]**;** however the timing of combined molecular targeted radiotherapy and immunotherapy warrants further investigation. 

## 5. Conclusions

These data demonstrate that the combined effects of FAP-targeted radiotherapy and checkpoint inhibitor therapy are highly tumor- and context-dependent but have the potential to provide increased anticancer efficacy compared with either treatment alone. Further, there is heterogeneity in response to combined therapy, which provides an opportunity to study the mechanism of these different responses. Our data also demonstrate that ^177^Lu-FAPI-04 is an effective anticancer agent in murine models of melanoma and lung cancer and has immunomodulatory effects mediated through macrophage populations. Future studies of the mechanism of these changes will provide insight as to how we can manipulate cellular and microenvironmental conditions to augment the response to therapy and ultimately translate it into an efficacious combined regimen for clinical use. 

## Figures and Tables

**Table 1:** Cell populations were defined based on the following markers.

Cell Type	Gating Strategy
B cells	CD3^–^/CD19^+^
Dendritic cells	CD3^–^/CD19^–^/CD11c^+^/MHCII^+^
Conventional DC lineage 1	CD3^–^/CD19^–^/CD11c^+^/MHCII^+^/XCR1^+^/CD11b^–/low^
Conventional DC lineage 2	CD3^–^/CD19^–^/CD11c^+^/MHCII^+^/XCR1^–^/CD11b^+^
Natural killer cells	CD3^–^/CD19^–^/CD11b^+^/Ly-6g^–^/NK1.1^+^
Monocytes	CD3^–^/CD19^–^/CD11b^+^/Ly-6g^–^/Ly-6c^+^
Neutrophils	CD3^–^/CD19^–^/CD11b^+^/Ly-6g^+^
Eosinophils	CD3^–^/CD19^–^/CD11b^+^
Macrophages (M1)	CD80^+^/CD11b^+^
Macrophage (M2)	CD11B^+^/CD206^+^

## Data Availability

Data are available upon request.

## Supplementary Materials

The following supporting information can be downloaded at: https://www.mdpi.com/article/10.3390/cancers14194575/s1, Figure S1: Average tumor growth at each time point for each treatment group in animals implanted with (**A**) melanoma and (**B**) lung cancer, respectively.; Figure S2: Representative flow gating graphs for the (**A**) dendritic lineage and (**B**) myeloid lineage cell populations analyzed in this study.
