# Supplementary material for: Evaluating the Combined Anticancer Response of Checkpoint Inhibitor Immunotherapy and FAP-Targeted Molecular Radiotherapy in Murine Models of Melanoma and Lung Cancer
Source: Cancers (Basel). 2022 Sep 21;14(19):4575. doi: 10.3390/cancers14194575 (PMC9559475; doi:10.3390/cancers14194575)
Supplement: Supplementary file 1 [file cancers-14-04575-s001.zip › cancers-1894067-supplementary.pdf]

Supplementary Materials

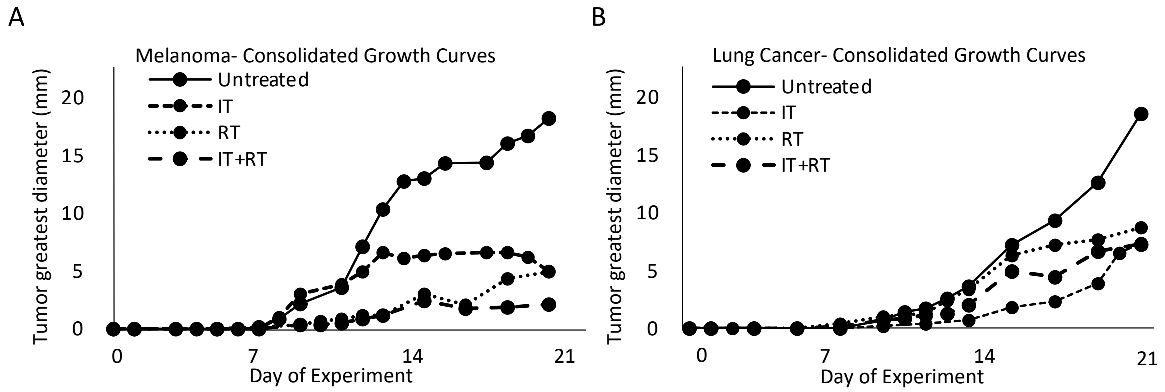

Supplemental Figure S1. Average tumor growth at each time point for each treatment group in animals implanted with (**A**) melanoma and (**B**) lung cancer, respectively.

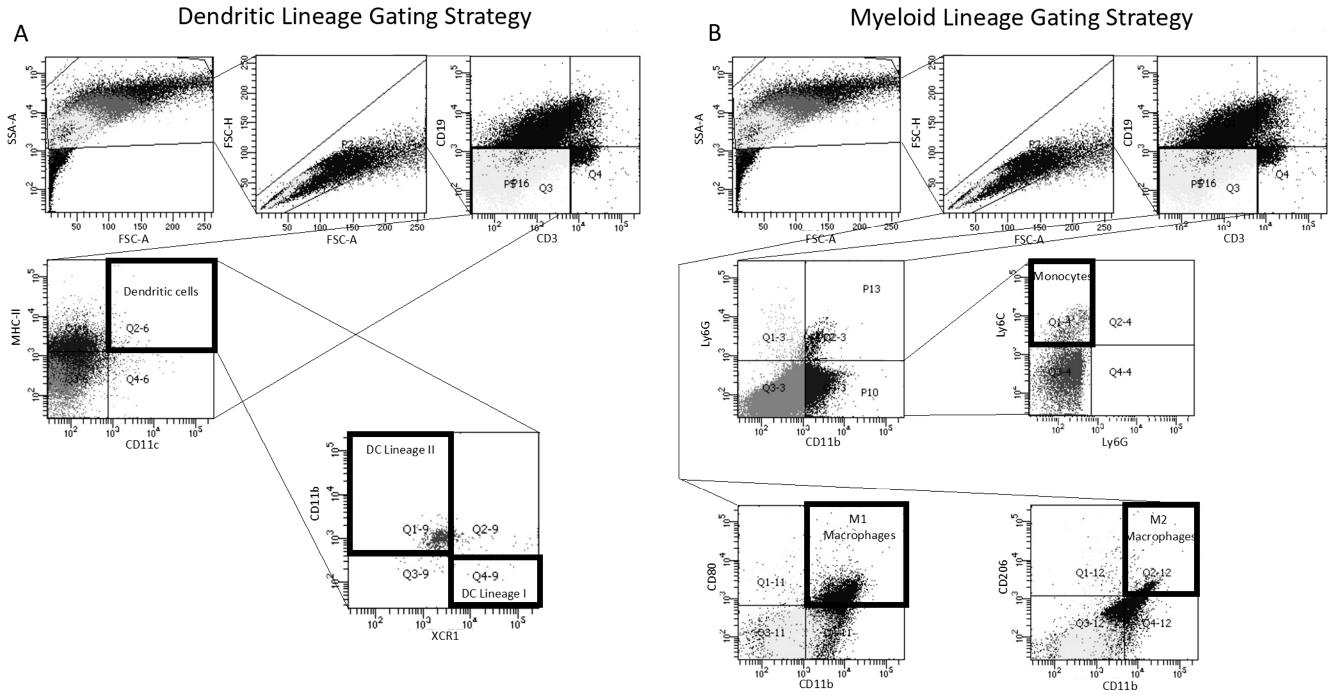

Supplemental Figure S2. Representative flow gating graphs for the (**A**) dendritic lineage and (**B**) myeloid lineage cell populations analyzed in this study.
